# Gene mutations associated with fertilization failure after *in vitro* fertilization/intracytoplasmic sperm injection

**DOI:** 10.3389/fendo.2022.1086883

**Published:** 2022-12-16

**Authors:** Yamei Xue, Xiaohong Cheng, Yuping Xiong, Kun Li

**Affiliations:** ^1^ Assisted Reproduction Unit, Department of Obstetrics and Gynecology, Sir Run Run Shaw Hospital, School of Medicine, Zhejiang University, Hangzhou, China; ^2^ Institute for Reproductive Health, School of Pharmacy, Hangzhou Medical College, Hangzhou, China; ^3^ Zhejiang Provincial Laboratory of Experimental Animal’s & Nonclinical Laboratory Studies, Hangzhou Medical College, Hangzhou, China

**Keywords:** sperm, fertilization, assisted reproductive technology (ART), *in vitro* fertilization (IVF), intracytoplasmic sperm injection (ICSI), oocyte, fertilization failure, gene mutation

## Abstract

Fertilization failure during assisted reproductive technologies (ART) is often unpredictable, as this failure is encountered only after in vitro fertilization (IVF) and intracytoplasmic sperm injection (ICSI) have been performed. The etiology of fertilization failure remains elusive. More and more mutations of genes are found to be involved in human fertilization failure in infertile patients as high throughput sequencing techniques are becoming widely applied. In this review, the mutations of nine important genes expressed in sperm or oocytes, *PLCZ1, ACTL7A, ACTL9, DNAH17, WEE2, TUBB8, NLRP5, ZP2*, and *TLE6*, were summarized and discussed. These abnormalities mainly have shown Mendelian patterns of inheritance, including dominant and recessive inheritance, although de novo mutations were present in some cases. The review revealed the crucial roles of each reported gene in the fertilization process and summarized all known mutations and their corresponding phenotypes. The review suggested the mutations might become promising targets for precision treatments in reproductive medicine. Moreover, our work will provide some helpful clues for genetic counseling, risk prediction, and optimizing clinical treatments for human infertility by supplying the useful and timely information on the genetic causes leading to fertilization failure.

## Introduction

Fertilization is a fundamental process of development and a hallmark event of sexual reproduction. In the process of fertilization, a sperm and a mature oocyte fuse to form a diploid zygote that will subsequently develop into a new life. Under physiological conditions, sperm pass through the vagina and uterus to migrate toward the ampulla of the oviduct. During this transit, sperm acquire the ability to fertilize eggs through capacitation. Capacitated sperm transverse the corona radiata, penetrate the zona pellucida, bind the egg, and reach the perivitelline space. Finally, the sperm membrane and the oolemma fuse to lead to a series of changes, including the exocytosis of cortical granules, completion of the oocyte meiosis, and formation of maternal and paternal pronucleus (1, 2). Successful fertilization is assessed by the presence of the second polar body and two pronuclei.

In the treatment of *in vitro* fertilization (IVF), approximately 20% of couples with normospermia have low fertilization rates (defined as < 25%), and 5-15% have total fertilization failure (TFF) (3). In an earlier study, in 1980, Trounson AO et al. first reported an investigation into the use of IVF as a diagnostic procedure for patients with long-term infertility of unknown cause (idiopathic infertility) (4). The results indicated that fertilization failure was one of the primary causes of infertility. Barlow P et al. evaluated clinical male and female factors that might be involved in the occurrence of fertilization failure in IVF and found three main factors, including the male factors, few oocytes retrieved, and low-quality oocytes (5). The advent of intracytoplasmic sperm injection (ICSI) in 1991 has become the gold standard technique to treat male factor infertility. It was developed to allow egg-sperm fusion to bypass the natural barriers around oocytes, such as corona radiata, zona pellucida, and the oolemma (6–9). The use of ICSI technology was initially limited to serving those presenting with oligo-, astheno-, and terato-zoospermia or those with a history of unexplained TFF, and later it was also extensively used in non-male factor infertility (10). Although a drastic improvement in fertilization rate was observed in ICSI, there are still 1-5% of ICSI cycles with TTF (11). Some couples have even experienced recurrent fertilization failure after attempting ICSI for unknown reasons, despite having sperm with normal morphology, motility, and concentration (12). Fertilization failure in IVF/ICSI treatment is a most frustrating experience for patients as well as for IVF physicians.

Identification of the essential gene mutations is very important not only for understanding the mechanisms underlying fertilization failure at molecular levels but also for the treatment and diagnosis of infertile couples. Recent advances in molecular genetics have drastically accelerated the identification of novel genes responsible for fertilization. High throughput sequencing techniques have been successfully used to identify pathogenic genes related to human fertilization failure in a large individual population. Clinical researchers have attributed at least some cases of fertilization failure to the loss of function of one or more genes.

In this review, we focus on the gene mutations that can contribute to the fertilization failure of IVF/ICSI attempts and summarize the recently hereditary findings. We aim to provide useful and timely information on the genetic causes and the biomarkers of infertility for patients.

## Methods

### Search strategy

A computerized literature search was conducted for all publications in PubMed/Medline until December 2021 using the following MeSH or keyword terms: “Fertilization failure” or “Failed fertilization” or “Poor fertilization” or “oocyte activation deficiency” AND “*In vitro* fertilization” or “IVF” or “Intracytoplasmic sperm injection” or “ICSI” AND “Whole exome sequencing” or “WES” or “Next generation sequencing” or “NGS” or “Sanger sequencing” AND “genetic” or “gene” or “Mutation” or “Variant”. The search strategy is shown in Supplementary Table S1.

### Study selection

The title, abstract, and full texts of the retrieved articles were reviewed, and the relevant articles were included. Only English-language studies or articles in other languages, but with a detailed abstract in English were enrolled. In addition, in the present systematic scoping review, we have opted to focus on the studies that discussed specific gene mutations associated with poor or failed fertilization in IVF/ICSI treatments and provided strong and convincing evidence for the roles of the genes in the corresponding phenotypes. We did not include articles regarding the association between single nucleotide polymorphisms and fertilization failure after IVF/ICSI because we believe that these studies may have a low level of evidence. Searching and screening of the articles were separately performed by two authors.

### Data extraction

The details of the enrolled articles, including author, year of publication, number of individuals studied, identified genes, the identified cDNA and amino acid alterations, and fertilization results of IVF/ICSI attempts are shown in **Table 1**.

**Table 1:** Details of included studies on gene mutations that lead to poor and failed fertilization.

Author, year	Number of patients studied (n)			Geographical origin	Identified genes	Identified cDNA alteration (Amino acid alteration)	IVF/ICSI cycles	Oocytes retrieved	Mature oocytes	Fertilized oocytes (%) #	References
	Included	Unrelated^c^	Newly tested								
Escoffier J et al., 2016	2	1	2	Tunisia	*PLCZ1*	c.1465A>T(p.Ile489Phe)	P1: ICSI = 2 P2: ICSI = 1	9, 9 14	4, 7 8	0 (0), 0 (0) 0 (0)	(13)
Torra-Massana M et al., 2019	37	37	13	Spain	*PLCZ1*	P1: c.590G>A (p.R197H) P2: c.698A>T(p.H233L) P3: c.972_973 delAG (p.V326K fs∗25) P4: c.1499C>T (p.S500 L) P5: c.1499C>T (p.S500 L) P6: c.671T>C (p.L224P) P7: c.1499C>T (p.S500 L) P8: c.1499C>T (p.S500 L) P9: c.1499C>T (p.S500 L) P10: c.360C>G (p.I120M) P11: c.1499C>T (p.S500 L) P12: c.1499C>T (p.S500 L) P13: c.1499C>T (p.S500 L)	P1: ICSI=1 P2: ICSI=2 P3: ICSI=1 P4: ICSI=4 P5: ICSI=6 P6: ICSI=2 P7: ICSI=1 P8: ICSI=1 P9: ICSI=3 P10: ICSI=2 P11: ICSI=3 P12: ICSI=1 P13: ICSI=4	NA	7 2, 5 5 11, 2, 3, 3 1, 4, 3, 8, 5, 7 5,3 7 4 6, 12, 13 10, 8 3, 3, 8 7 2, 4, 4, 10	0(0) 0(0), 0(0) 0(0) 0(0), 0(0),0(0), 2(66.7) 0(0), 0(0),2(66.7), 0(0),0(60.0), 0(0) 0(0), 1(33.3) 1(14.3) 0(0) 0(0),1(8.3), 0(0) 0(0), 0(0) 1(33.3), 1(33.3), 1(12.5) 1(14.3) 0(0), 0(0),0(0), 5(50.0)	(14)
Dai J et al., 2019	10	10	3	China	*PLCZ1*	P1: c.C588A (p.C196X) P2: c.T1048C (p.S350P) P3: c.C736T (p.L246F)	P1: ICSI=1, ICSI+AOA=1 P2: ICSI=1, ICSI+AOA=1 P3: ICSI=1, ICSI+AOA=1	20, 20 18, 14 14, 7	17,18 15, 6 13, 4	0(0), 16 (88.9) 0(0), 2(33.3) 1(7.7), 3(75.0)	(15)
Yan Z et al., 2020	14	14	5	China	*PLCZ1*	P1: c.588C>A p.C196^∗^ P2: c.588C>A p.C196*, c.830T>C p.L277P P3: c.1129_1131delAAT p.N377del, c.1733T>C p.M578T P4: c.1151C>T p.A384V P5: c.570+1G>T p.V189Cfs^∗^12, c.1344A>T p.K448N	P1: ICSI= 3 P2: ICSI= 2 P3: ICSI= 2 P4: ICSI= 1 P5: ICSI= 2	NA	29^$^ 15^$^ 24^$^ 25^$^ 24^$^	3(10.3) 0(0) 0(0) 5(20.8) 0(0)	(16)
Yuan P et al., 2020	2	2	2	China	*PLCZ1*	P1: c.1259C>T (p.P420L), c.1733T>C (p.M578T) P2: c.1727T>C (p.L576P)	P1: IVF=1, IVF+ICSI=1 P2: IVF+ICSI=2, ICSI=1	8, 4 6, 7, 14	6, 4 6, 6, 13	0(0),0(0) 1(16.7), 2(33.3), 6(46.2)	(17)
Yuan P et al., 2020	1	1	1	China	*PLCZ1*	c.1658 G>C (p. R553P)	ICSI = 3	NA	22, 4, 24	0(0),0(0),0(0)	(18)
Mu J et al., 2020	4	4	4	China	*PLCZ1*	P1: c.588C>A (p.Cys196∗) P2: c.588C>A (p.Cys196∗), c.1259C>T (p.Pro420Leu) P3: c.590G>A (p.Arg197His) P4: c.972_973delAG p.Thr324fs, c.1234delA (p.Arg412fs)	P1: IVF=1, ICSI=4, ICSI+AOA=1 P2: IVF=1, ICSI=1, ICSI+AOA=1 P3: ICSI=3, ICSI+AOA=1 P4: ICSI=5, ICSI+AOA=1	10,7,6,2,1,4 19, 16,14 5,13,25,19 12,6,5,6,1,28	10,7,6,2,1,2 17,12,8 3,8,21,10 12,6,2,2,0,26	2(20.0),1(14.3),1(16.7),0(0),0(0),2(100) 1(5.9),0(0),8(100) 2(66.7),2(25.0),6(28.6),9(90.0) 0(0),0(0),0(0),0(0), 0(0),8(30.8)	(19)
Wang F et al., 2020	4	4	1	China	*PLCZ1*	c.588C>A (p.Cys196∗)	IVF= 1; ICSI=1; ICSI+AOA= 2	16,13,9,2	NA,10,8,2	0(0),0(0),3(37.5),2(100)	(20)
Dai J et al., 2018	24	22	5	China	*WEE2*	P1: c.G585C (p.Lys195Asn) P2: c.1228C>T (p.Arg410Trp) P3: c.1006_1007dup (p.His337Tyrfs*24) P4: c.1006_1007dup (p.His337Tyrfs*24), c.1136-2A>G (p.Asp380Leufs*39) P5: c.1006_1007dup (p.His337Tyrfs*24)	P1: IVF=1, ICSI=1 P2: IVF=1, ICSI=1 P3: IVF=1, ICSI=1, ICSI+AOA=1 P4: IVF+ICSI=1, ICSI+AOA=1 P5: IVF+ICSI=1	9, 12 18, 21 13, 10,10 15, 8 21	7, 7 18, 16 10, 9, 8 11, 8 17	0(0),0(0) 2 (11.1),1(6.3) 0(0),0(0),0(0) 0(0),0(0) 0(0)	(21)
Sang Q et al., 2018	4	4	4	China	*WEE2*	P1: c.700G>C (p.Asp234His) P2: c.1473dupA (p.Thr493Asnfs*39) P3: c.220_223delAAAG (p.Glu75Valfs*6) P4: c.1006_1007insTA (p.His337Tyrfs*24)	P1: ICSI = 2 P2: ICSI = 1 P3: IVF = 1, ICSI = 2 P4: ICSI = 1	3 ^a^ 11 19 ^a^ 20	3 8 18 20	0(0) 0(0) 0(0) 0(0)	(22)
Zhao S et al., 2018	90	90	4	China	*WEE2*	P1: c.293_294insCTGAGACACCAGCCCAACC (p.Pro98Pro fsX2) P2: c.1576T>G (p.Tyr526Asp) P3: c.991C>A (p.His331Asn), c.1304_1307delCCAA (p.Thr435Met fsX31) P4: c.341_342 del AA (p.Lys114Asn fsX20), c.864G>C (p.Gln288His) P5: c.1A>G (p.0)?, c.1261G>A (p.Gly421Arg)	P1: IVF=1, ICSI=2, IVF+ICSI=1 P2: IVF=1, ICSI=1 P3: IVF=1, ICSI=1 P4: IVF=1, ICSI=1 P5: IVF=1, ICSI=1	5, 6, 5, 5 18, 11 15,12 12, 10 6, 21	NA	1(20.0), 0(0), 0(0), 0(0) 1(5.6), 0(0) 0(0), 0(0) 0(0), 0(0) 0(0), 0(0)	(23)
Yang X et al., 2019	1	1	1	China	*WEE2*	c.619C>T (p.R207C)	Rescue ICSI =1, ICSI+AOA = 1	23,16	NA, NA	0(0), 0(0)	(24)
Zhou X et al., 2019	17	17	1	China	*WEE2*	c.598C>T (p.Arg200Ter), c.1319G>C (p.Trp440Ser)	IVF=1, ICSI =1	15, 27	NA, 22	0(0),0(0)	(25)
Tian Y et al., 2020	1	1	1	China	*WEE2*	c.1535+3A>G(p.)?, c.946C > T (p. Leu316Phe)	IVF= 1; ICSI=2; ICSI+AOA= 1	15,NA,NA,NA	NA,8,9,11	0(0),2(25.0);0(0);0(0)	(26)
Wang A et al., 2021	6	6	1	China	*WEE2*	c.625G>T(p.E209*), c.759-2A>G (p.)?	Rescue ICSI =1, ICSI+AOA = 1	8, 8	4,8	0(0),0(0)	(27)
Jin J et al., 2021	31	31	3	China	*WEE2*	P1: c.220_223delAAAG (p.Glu75Valfs*6), c.G585C (p.Lys195Asn) P2: c.115_116insT (p.Gln39Leufs*5), c.C1459T (p.Arg487Trp) P3: c.756_758delTGA (p. Asn252Lysfs*316), c.1006_1007insTA (p. His337Tyrfs*24)	P1: IVF=1, ICSI=1 P2: IVF=1, ICSI=1 P3: IVF=1, ICSI=2	14,14 19,17 19,18,14	14,11 19,16 12,16,9	0(0),0(0), 0(0), 3(18.8) 2(16.7), 0(0),0(0)	(28)
Yang P et al., 2021	115	115	4^&^	China	*TUBB8*	P1: c.629T>A (p.I210K) P2: c.938C>T (p.A313V) P3: c.1130T>C (p.L377P) P4: c.1203_1204insCT (p.G402Lfs*15)	P1: NA P2: NA P3: NA P4: NA	NA NA NA NA	NA NA NA NA	Fertilization failure Fertilization failure Fertilization failure Fertilization failure	(29)
Chen B et al., 2016	1	1	1	China	*TUBB8*	c.209 C > T (p.P70L)	IVF=1, ICSI=1	NA, 25	NA, 20	0 (0),2 (10.0)	(30)
Zhao L et al., 2020	2	2	2	China	*TUBB8*	P1: c.260C>T (p.P87L) P2: c.716G>C (p.C239S)	3 cycles 2 cycles	44^$^ 17^$^	NA 12	9 (NA) 2 (16.7)	(31)
Chen B et al.	4	4	4	China	*TUBB8*	P1: c.322G>A (p.E108K) P2: c.1270C>T (p.Q424*) P3: c.10A>C (p. I4L) P4: c.1228G>A (p. E410K)	2 cycles 3 cycles 3 cycles 2 cycles	27^$^ 40^$^ 26^$^ 22^$^	20 19 21 7	2 (10.0) 0 (0) 4 (19.0) 0 (0)	(32)
Li M et al., 2020	1	1	1	China	*NLRP5*	c.1598G>C (p.Arg533Pro), c.1919T>G (p.Leu640Arg)	IVF =1, ICSI = 1	13, 11	NA, 8	0(0),0(0)	(33)
Maddirevula S et al., 2020	1	1	1	Saudi Arabia	*NLRP5*	c.2274_2275del(p.Trp759Aspfs*4)	IVF+ICSI=1, ICSI=1	28,18	NA, 6	0(0),0(0)	(34)
Wang J et al., 2021	1	1	1	China	*ACTL7A*	c.463C>T (p.Arg155Ter), c.1084G>A (p.Gly362Arg)	ICSI=1, ICSI+AOA =1	11, 13	11,13	0(0),10(76.9)	(35)
Dai J et al., 2021	21	21	3	China	*ACTL9*	P1: c.1034C>T (p.Ser345Leu) P2: c.1138G>T (p.Val380Leu) P3: c.1209C>G (p.Tyr403Ter)	P1: IVF =1, ICSI =1 P2: ICSI =1 P3: IVF =1, ICSI =2	6, 14 12 11, 20, 14	4,11 10 11,19,12	0(0),0(0) 0(0) 0(0),1(5.2),2(16.7)	(36)
Dai C et al., 2018	2	2	2	China	*ZP2*	P1: c.1695-2A>G (p.C566H fs*5) P2: c.1691_1694dup (p.C566W fc*5)	P1: Half-ICSI=1 P2: IVF=1, Half-ICSI=1	16 17, 25	NA	8(100) ^b^ 0(0), 12 (80.0) ^b^	(37)
Alazami A M et al., 2015	3	2	2	Saudi Arabia	*TLE6*	c.1529C > A:p.S510Y	P1: ICSI=10 P2: ICSI=2	58 19	NA NA	3 (5.2) 0(0	(38)
Lin J et al., 2020	403	403	3	China	*TLE6*	P1: c.1226G>A(p.Arg409Gln) P2: c.1621G>A(p.Glu541Lys) P3: c.388G>A (p.Asp130Asn), c.1507G>A (p.Val503Ile)	P1: ICSI = 2 P2: IVF =1, ICSI = 4 P3: IVF =1, ICSI = 2	8,6 NA, 2, 11, 8, 8 8, 8, 14	NA	0(0),0(0) 1(NA),1(50.0), (63.6),1(12.5),3(37.5) 4(50.0),7(87.5),11(78.6)	(39)
Jia M et al., 2021	1	1	1	China	*DNAH17*	c.1048C>T(p.Arg350*); c.3390G>A(p.Met1130Ile)	IVF=1(rescue ICSI =1); ICSI+AOA=1	20,15	13,10	0(0),0(0)	(40)

a: indicated the total number of oocytes retrieved.

b: All mature oocytes were divided into two groups for IVF and ICSI. None of the oocytes in the IVF groups were fertilized. In ICSI group, all 8 (100%) oocytes from P1 and 12 of the 15 (80%) from P2 were fertilized.

c: indicated the number of unrelated patients among all included patients.

^$^: indicated the total number of oocytes in all cycles.

^&^: indicated the number of patients diagnosed with fertilization failure.

*: indicated stop codon (nonsense mutation).

^#^: indicated the number of fertilized oocytes (fertilization rate).

AOA, assisted oocyte activation; IVF, in vitro fertilization; ICSI, intracytoplasmic sperm injection; IVF+ICSI indicated that ICSI was performed on those oocytes that failed to fertilize in conventional IVF; ICSI+AOA indicated that AOA was performed on those oocytes that failed to fertilize in ICSI; P, patient; NA, not available.

## Results

**Figure 1:**
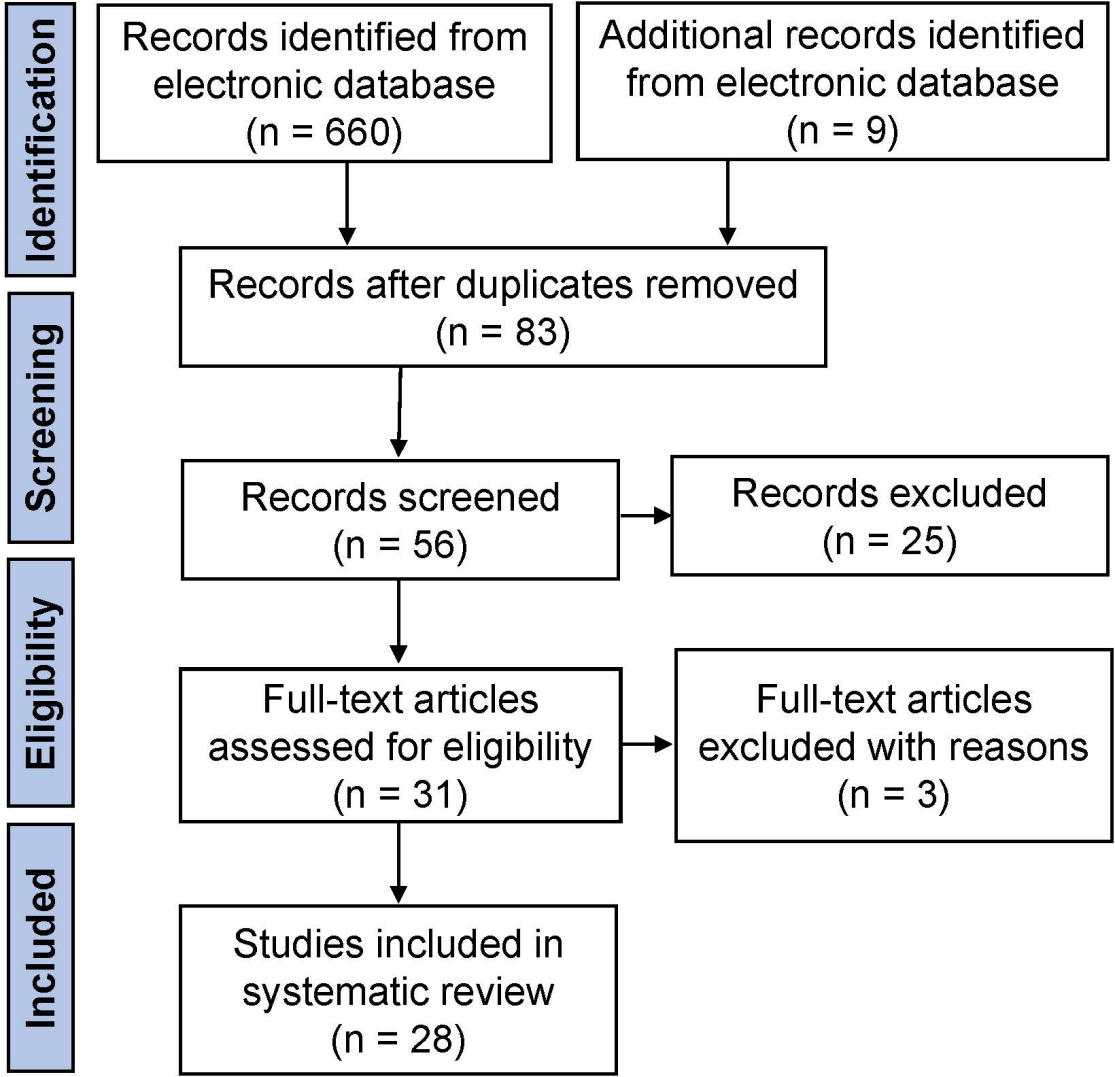
Workflow diagram of the literature review procedure.

**Figure 2:**
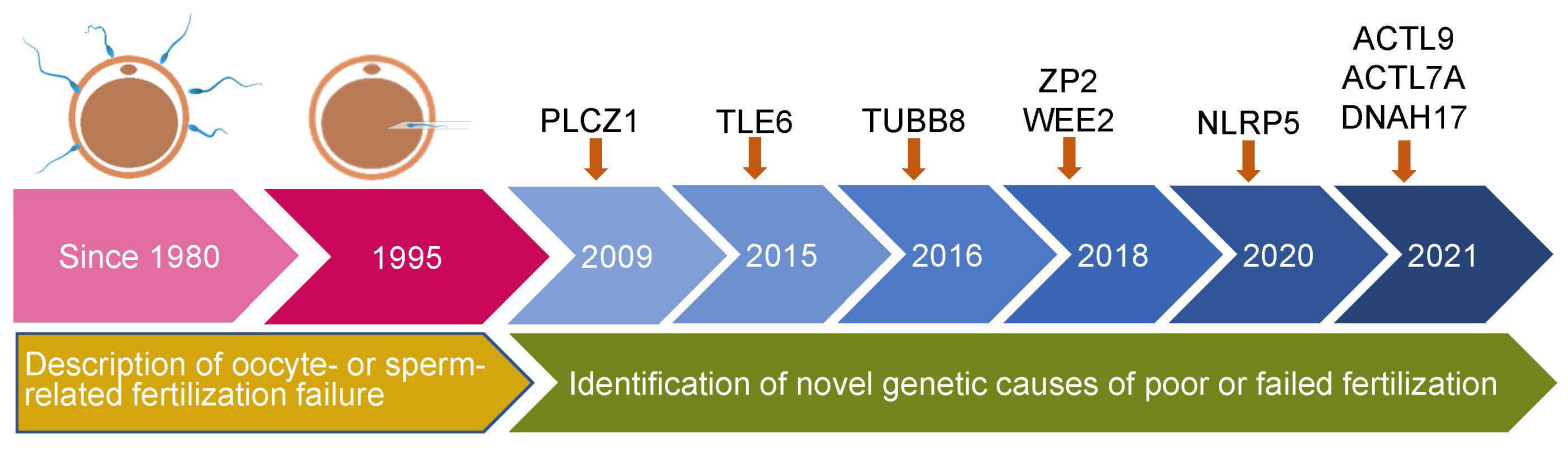
Timeline of discovery of mutations of genes that lead to poor and failed fertilization. PLCZ1, Phospholipase C zeta 1; WEE2, WEE1 Homolog 2; TUBB8, Tubulin beta 8 class VIII; NLRP5, NLR family pyrin domain containing 5; ACTL9, Actin-like 9; ACTL7A, Actin-like protein 7A; ZP2, Zona pellucida glycoprotein 2; TLE6, transducin-like enhancer of split 6; DNAH17, Dynein axonemal heavy chain 17.

The initial literature search yielded 669 articles from the electronic database, of which 83 studies finally remained after removing duplicates. According to the inclusion criteria, of the remaining papers, 28 studies were comprised in the current systematic scoping review. A flow diagram of the study is shown in **Figure 1**. The details about the studies included are listed in **Table 1**. In the past decades, 9 genes (PLCZ1, ACTL7A, ACTL9, DNAH17, WEE2, TUBB8, NLRP5, ZP2, and TLE6) have been reported to be causes of fertilization failure (**Figure 2**). **Table 2** presented all known mutations discovered in infertile patients with fertilization failure by WES and Sanger sequencing technologies. All mutations and their corresponding phenotypes were summarized in **Table 3**.

**Table 2:** Genetic mutations associated with poor or failed fertilization after IVF/ICSI by WES and Sanger sequencing technologies.

Identified genes	Full name	MIM number	cDNA alteration	Amino acid alteration	Exon	Mutation	Zygosity in affected individuals	References
*PLCZ1*	Phospholipase C zeta 1	608075	c.1465A>T	p.Ile489Phe	Exon 13	Missense	Homozygous	(13)
			c.360C>G	p.I120M	Exon 4	Missense	Heterozygous	(14)
			c.590G>A	p.R197H	Exon 6	Missense	Heterozygous	(14)
			c.671T>C	p.L224P	Exon 6	Missense	Heterozygous	(14)
			c.698A>T	p.H233L	Exon 6	Missense	Heterozygous	(14)
			c.972_973 delAG	p.V326K fs∗25	Exon 9	Frameshift	Heterozygous	(14)
			c.1499C>T	p.S500 L	Exon 13	Missense	Homozygous	(14)
			c.588C>A	p.C196*	Exon 6	Nonsense	Homozygous	(15, 16, 19, 20)
			c.T1048C	p.S350P	Exon 10	Missense	Homozygous	(15)
			c.C736T	p.L246F	Exon 7	Missense	Homozygous	(15)
			c.830T>C p.L277P	p.L277P	Exon 7	Missense	Homozygous	(16)
			c.1129_1131delAAT	p.N377del	Exon 10	Frameshift	Compound heterozygous	(16)
			c.1733T>C	p.M578T	Exon 14	Missense	Compound heterozygous	(16)
			c.1151C>T	p.A384V	Exon 10	Missense	Homozygous	(16)
			c.570+1G>T	p.V189Cfs^∗^12	Exon 5	Splicing	Compound heterozygous	(16)
			c.1344A>T	Heterozygous	Exon 12	Missense	Compound heterozygous	(16)
			c.1259C>T	p.P420L	Exon 11	Missense	Compound heterozygous	(17)
			c.1733T>C	p.M578T	exon 14	Missense	Compound heterozygous	(17)
			c.1727T>C	p.L576P	Exon 14	Missense	Homozygous	(17)
			c.1658 G>C	p. R553P	NM	Missense	Homozygous	(18)
			c.1259C>T	p.Pro420Leu	Exon 11	Missense	Heterozygous	(19)
			c.590G>A	p.Arg197His	Exon 6	Missense	Homozygous	(19)
			c.972_973delAG	p.Thr324fs	Exon 9	Frameshift	Compound heterozygous	(19)
			c.1234delA	p.Arg412fs	Exon 11	Frameshift	Compound heterozygous	(19)
*WEE2*	*WEE1 Homolog 2*	614084	c.G585C	p.Lys195Asn	Exon 3	Missense	Homozygous	(21)
			c.1228C>T	p.Arg410Trp	Exon 9	Missense	Homozygous	(21)
			c.1006_1007dup	p.His337Tyrfs*24	Exon 6	Frameshift	Compound heterozygous	(21, 22, 28)
			c.1136-2A>G	p.Asp380Leufs*39	IVS 7	Splicing	Compound heterozygous	(21)
			c.700G>C	p.Asp234His	Exon 4	Missense	Homozygous	(22)
			c.1473dupA	p.Thr493Asnfs*39	Exon 10	Frameshift	Homozygous	(22)
			c.220_223delAAAG	p.Glu75Valfs*6	Exon 1	Frameshift	Homozygous	(22)
			c.293_294insCTGAGACACCAGCCCAACC	p.Pro98Pro fsX2	Exon 1	Frameshift	Homozygous	(23)
			c.1576T>G	p.Tyr526Asp	Exon 11	Missense	Homozygous	(23)
			c.991C>A	p.His331Asn	Exon 6	Missense	Compound heterozygous	(23)
			c.1304_1307delCCAA	p.Thr435Met fsX31	Exon 9	frameshift	Compound heterozygous	(23)
			c.341_342 del AA	p.Lys114Asn fsX20	Exon 1	Frameshift	Compound heterozygous	(23)
			c.864G>C	p.Gln288His	Exon 5	Missense	Compound heterozygous	(23)
			c.1A>G	p.0?	Exon 1	Frameshift	Compound heterozygous	(23)
			c.1261G>A	p.Gly421Arg	Exon 9	Missense	Compound heterozygous	(23)
			c.619C>T	p.R207C	Exon 4	Missense	Homozygous	(24)
			c.598C>T	p.Arg200Ter	NM	Nonsense,	Compound heterozygous	(25)
			c.1319G>C	p.Trp440Ser	NM	Missense	Compound heterozygous	(25)
			c.625G>T	p.E209*	Exon 4	Nonsense	Compound heterozygous	(27)
			c.759-2A>G	p.?	Exon 5	Splicing	Compound heterozygous	(27)
			c.220_223delAAAG	p.Glu75Valfs*6	Exon 1	Frameshift	Compound heterozygous	(28)
			c.G585C	p.Lys195Asn	exon 3	Missense	Compound heterozygous	(28)
			c.115_116insT	p.Gln39Leufs*5	Exon 1	Frameshift	Compound heterozygous	(28)
			c.C1459T	p.Arg487Trp	Exon 10	Missense	Compound heterozygous	(28)
			c.756_758delTGA	p. Asn252Lysfs*316	Exon 4	Frameshift	Compound heterozygous	(28)
			c.1535+3A>G	p.?	IVS10	Splicing	Compound heterozygous	(26)
			c.946C > T	p. Leu316Phe	Exon 6	Missense	Compound heterozygous	(26)
*TUBB8*	Tubulin beta 8 class VIII	616768	c.629T>A	p.I210K	Exon 4	Missense	Heterozygous	(29)
			c.938C>T	p.A313V	Exon 4	Missense	Heterozygous	(29)
			c.1130T>C	p.L377P	Exon 4	Missense	Heterozygous	(29)
			c.1203_1204insCT	p.G402Lfs*15	Exon 4	Frameshift	Heterozygous	(29)
			c.613G>A	p.E205K	Exon 4	Missense	Heterozygous	(29)
			c.209 C > T	p.P70L	Exon 3	Missense	Homozygous	(30)
			c.260C>T	p.P87L	Exon 3	Missense	Heterozygous	(31)
			c.716G>C	p.C239S	Exon 4	Missense	Heterozygous	(31)
			c.322G>A	p.E108K	Exon 4	Missense	Homozygous	(32)
			c.1270C>T	p.Q424*	Exon 4	Nonsense	Compound heterozygous	(32)
			c.10A>C	p. I4L	Exon 1	Missense	Heterozygous	(32)
			c.1228G>A	p. E410K	Exon 4	Missense	Compound heterozygous	(32)
*NLRP5*	NLR family pyrin domain containing 5	609658	c.1598G>C	p.Arg533Pro	Exon 7	Missense	Compound heterozygous	(33)
			c.1919T>G	p.Leu640Arg	Exon 7	Missense	Compound heterozygous	(33)
			c.2274_2275del	p.Trp759Aspfs*4	NM	Frameshift	Homozygous	(34)
*ACTL9*	Actin like 9	619251	c.1034C>T	p.Ser345Leu	Exon 1	Missense	Homozygous	(36)
			c.1138G>T	p.Val380Leu	Exon 1	Missense	Homozygous	(36)
			c.1209C>G	p.Tyr403Ter	Exon 1	Nonsense	Homozygous	(36)
*ACTL7A*	Actin-like protein 7A	604303	c.463C>T	p.Arg155Ter	Exon 1	Nonsense	Compound heterozygous	(35)
			c.1084G>A	p.Gly362Arg	Exon 1	Missense	Compound heterozygous	(35)
*ZP2*	Zona pellucida glycoprotein 2	182888	c.1695-2A>G	p.C566H fs*5	Exon 4	Missense	Homozygous	(37)
			c.1691_1694dup	p.C566W fc*5	Exon 4	Frameshift	Homozygous	(37)
*TLE6*	transducin-like enhancer of split 6	612399	c.1226G>A	p.Arg409Gln	Exon 13	Missense	Homozygous	(39)
			c.1621G>A	p.Glu541Lys	Exon 17	Missense	Homozygous	(39)
			c.388G>A	p.Asp130Asn	Exon 7	Missense	Compound heterozygous	(39)
			c.1507G>A	p.Val503Ile	Exon 15	Missense	Compound heterozygous	(39)
			c.1529C > A	p.S510Y	NM	Splicing	Homozygous	(38)
*DNAH17*	Dynein axonemal heavy chain 17	610063	c.1048C>T	p.Arg350*	Exon 7	Nonsense	Compound heterozygous	(40)
			c.3390G>A	p.Met1130Ile	Exon 22	Missense	Compound heterozygous	(40)

NM, not mentioned.

*, indicated stop codon (nonsense mutation).

**Table 3:** Summary of gene mutations associated with poor or failed fertilization after IVF/ICSI and ART possible decision.

Gene		Phenotype in mutations				Mode of inheritance	MOAT^#^	ART possible decision
		Fertilization in IVF, %	Fertilization in ICSI, %	Fertilization in ICSI+AOA, %	References				
Sperm-related	*PLCZ1*	0-20.0%	0-66.7%	30.8-100%	(13–20)	AR	–	ICSI+AOA	
	*ACTL7A*	NA	0%	76.9%	(35)	AR	NA	ICSI+AOA	
	*ACTL9*	0%	0-16.7%	100%	(36)	AR	–	ICSI+AOA	
	*DNAH17*	0%	0%	NA	(40)	AR	NA	ICSI+AOA or Donor sperm	
Oocyte-related	*WEE2*	0-20.0%	0-25.0%	0%	(21–28)	AR	NA	Donor oocytes	
	*TUBB8*	0-19.0%	0-19.0%	NA	(29–32)	AD, AR, *de novo*, incomplete dominance, unknown	NA	ICSI+AOA or Donor oocytes	
	*NLRP5*	0%	0%	NA	(33, 34)	AR	NA	ICSI+AOA or Donor oocytes	
	*TLE6*	0-50%	0-87.5%	NA	(38, 39)	AR	NA	ICSI or Donor oocytes	
	*ZP2*	0%	80-100%	NA	(37)	AR	NA	ICSI	

# ‘-’ represents a negative result, which means the percentage of two-cells is less than 20%; ‘+’ represents a positive result, which means the percentage of two-cells is more than 90%.

IVF, in vitro fertilization; ICSI, intracytoplasmic sperm injection; ICSI+AOA indicated that AOA (assisted oocyte activation) was performed on those oocytes that failed to fertilize in ICSI; MOAT, mouse oocyte activation test; FR, fertilization rate; AD, autosomal dominant; AR, autosomal recessive; ART, assisted reproductive technology; NA, not available.

## Discussion

### Genetic alterations associated with poor or failed fertilization after IVF/ICSI attempts

Recent descriptive data obtained from whole exome sequencing (WES) and Sanger sequencing in humans have revealed gene mutations causing poor or failed fertilization after IVF/ICSI attempts (**Table 2**).

### PLCZ1

Intracellular Ca^2+^ oscillations are a remarkable signaling phenomenon observed during the process of mammalian fertilization (41–43). Sperm-specific phospholipase C (PLC) termed PLCzeta (PLCZ1) is widely considered to be the physiological stimulus responsible for generating Ca^2+^ oscillations (44, 45). As the smallest member of the PLC family (∼70 kDa in humans), PLCZ1 consists of a C-terminal C2 domain, four tandem Ca^2+^-binding EF-hand domains, and the X and Y catalytic domains (45, 46). Each domain plays a specific role in determining the function of sperm PLCZ1 as a trigger of oocyte activation and early embryonic development (46). PLCZ1 identified in sperm from fertile men usually expressed within the equatorial, acrosomal, and post-acrosomal regions of the head (17, 47, 48).

In 2009, a point mutation (H398P) in the PLCZ1 gene [MIM: 608075] was first discovered in a non-globozoospermic case linked to defects in the ability of the sperm to induce calcium oscillations in the oocyte following ICSI (49). To date, 24 PLCZ1 variants associated with poor or failed fertilization after IVF/ICSI attempts have been identified using Sanger sequencing and WES in infertile men. Segregation analysis and family pedigrees showed that it was an autosomal recessive mode of inheritance (13–16, 19, 20, 46, 50). It was indicated that these 24 reported mutationswithin the PLCZ1 gene include missense, frameshift, splicing, and nonsense that were localized in the C2 domain, EF-hand domains, and catalytic domains. The homozygous nonsense mutation c.588C>A (p.Cys196*), which maps to the catalytic domain, has been frequently reported (13, 14, 20, 50). Interestingly, Torra-Massana et al. reported a missense variant, c.1499C>T (p.S500L), located in the C2-domain of PLCZ1, which seems to be the most frequent mutation (19). This mutation was found in nine patients with poor or failed fertilization after ICSI (19).

The pathogenicity of these identified mutations and their possible effects on PLCZ1 protein were assessed through bioinformatics analysis and revealed that these important mutations probably weakened the stability of protein function (15, 19, 20, 46, 50). PLCZ1 protein structure consists of two main regions: the catalytic domain (X- and Y-domains) and the regulatory region (EF-hands and C2- domains), suggesting that the distinct locations of identified mutations may have different effects on the PLCZ1 protein function, which may lead to different phenotypes: total or partial fertilization failure (19, 20). Additionally, microinjection with the wild-type or mutant PLCZ1 cRNA into oocytes was performed to evaluate the effect of the identified mutation on protein function. Microinjection with the mutant cRNA into oocytes failed to activate oocytes to induce the formation of pronuclear, while the injection of wild-type cRNA could effectively do, indicating that these mutations have an adverse influence on protein function (13, 15, 16, 19, 20).

The histological examination performed in PLCZ1 knockout mice revealed that loss of PLCZ1 has no deleterious effects on spermatogenesis or quality parameters associated with the ability of the sperm to penetrate, bind and fuse with the egg (18). However, PLCZ1-null sperm failed to trigger Ca^2+^ oscillations in the egg *in vitro*. In addition, the incidence of polyspermy following IVF or *in vivo* fertilization increased significantly, indicating that PLCZ1-null sperm cannot induce Ca^2+^ oscillations, which is involved in the mechanism of preventing polyspermy (18).

### WEE2

WEE2 (WEE1 homolog 2, also known as WEE1B) [MIM: 614084] encodes a well-conserved oocyte-specific kinase which acts as an essential dual regulator of meiosis during prophase I and metaphase II by phosphorylating Tyr15 of the CDK1/cyclin B complex (M-phase promoting factor; MPF). In the GV stage of an oocyte, inhibition of WEE2 results in germinal vesicle breakdown (GVBD) and the resumption of meiosis. The WEE2 down-regulation leads to the failure of the MII stage exit and blockade of fertilization (51). WEE2 is predominantly expressed in the ovary of the rhesus macaque and weakly detectable in the testis, but not detected in any of the somatic tissues (52). Within the ovary, the expression of WEE2 persists in the germinal vesicle and cytoplasm of metaphase I and normal MII oocytes and reaches the highest level in preovulatory follicles (52).

In recent years, more and more evidence shows that WEE2 gene mutations may lead to fertilization failure and female infertility. Eight studies have so far reported a total of 27 mutations of WEE2 in patients with fertilization failure or poor fertilization, including 12 missense mutations, 10 frame-shift mutations, three splice-site mutations, and two nonsense mutations (21–24, 26–28, 53). The homozygous frameshift mutation c.1006_1007dup (p.His337Tyrfs*24) was repeatedly reported in three articles (27, 28, 53). Furthermore, in the study by Dai J et al., it was detected in four of nine patients, indicating that the incidence of this mutation is relatively high (53). These mutations follow an autosomal recessive pattern and proved to be truncated or loss of function, thereby reducing the protein level and disturbing the phosphorylation of WEE2 (22–24, 26–28, 53). In all reports describing female infertility linked to WEE2 mutations, nearly all the retrieved oocytes from affected individuals failed to form two pronuclei after IVF/ICSI attempts. Moreover, ICSI-AOA could not rescue fertilization failure (22, 26, 53). Interestingly, the researchers achieved phenotypic rescue by injection of WEE2 cRNA into affected oocytes, as indicated by the meiotic resumption, extrusion of the second polar body, and formation of pronuclei, embryonic development (28).

### TUBB8

TUBB8 (Tubulin beta 8 class VIII) [MIM:616768] encodes a highly conserved β-tubulin isotype that only exists in primates. Human β-tubulin consisted of nine isotypes, including TUBB1, TUBB2A, TUBB2B, TUBB3, TUBB4A, TUBB4B, TUBB5, TUBB6, and TUBB8 (25). TUBB8 accounts for the majority of all the β-tubulin isotype expressed in human oocytes and early embryos and play a key role in oocyte meiotic spindle assembly (54, 55). Most mutations in TUBB8 reported during recent years were associated with the maturation arrest of human oocytes and pre-implantation embryonic development abnormalities, while A few mutations in TUBB8 are responsible for poor or failed fertilization (29, 30, 32, 56).

Since 2016, 12 TUBB8 mutations associated with poor or failed fertilization have been identified using WES and Sanger sequencing. These TUBB8 mutations are paternally hereditary as an autosomal dominant, recessive inheritance or arise from *de novo*. It was indicated that 12 reported mutations in the TUBB8 gene include missense, nonsense, and frameshift. The effect of the TUBB8 mutations on microtubule dynamics, α/β tubulin heterodimeric assembly, and spindle assembly in mouse and human oocytes was evaluated by microinjection of the corresponding cRNAs (54, 55). The expression of wild-type and mutant forms of TUBB8 was measured in cultured cells (31, 54, 55). These mutations were found to interfere with α/β-tubulin heterodimeric folding, microtubule assembly, and stability, or to affect the interaction of microtubules with kinesin and potentially other microtubule-associated proteins (MAPs). The association of TUBB8 mutations with poor or failed fertilization may be due to that it results in abnormalities in the oocytes morphologically identifiable as MII, causing failure of the second polar body extrusion or zygote cleavage. The pathogenicity of these TUBB8 mutations was usually evaluated by a variety of methods, including spindle morphological observation, proband gene detection, pedigree segregation analysis, and *in vitro* experiments. However, it should be noted that due to a high prevalence of mutations in TUBB8, some of the mutations were only observed in the proband, and further *in vitro* functional verification was not carried out. Therefore, the causal relationship between these TUBB8 mutations and observed phenotypes needs to be further studied.

### NLRP5

NLRP5 (NLR family, Pyrin domain containing 5) is a maternal effect gene [MIM: 609658], originally identified in the mouse, which is exclusively expressed in the oocyte (57). The NLRP5-encoded protein belongs to the NALP protein family. Members of the NALP protein family typically consist of an amino N-terminal pyrin domain (PYD), a NACHT domain, and a carboxyl C-terminal leucine-rich repeat (LRR) region. PYD is a protein-protein interaction module, which has been identified in multiple human proteins involved in stress signaling pathways (58). The NACHT domain is presumed to bind ATP (59). LRR region is predicted to provide support for the formation of protein-protein interactions (60). As an oocyte-selective gene, NLRP5 plays a vital role in embryogenesis in mice (61), bovines (62), rhesus macaque monkeys (63), and humans (64). NLRP5 knockout female mice are infertile due to embryo development arrest at the 2-cell stage, although follicular development, oocyte maturation, and fertilization are fairly normal (61). In rhesus macaque, the NLRP5 knockout embryos had a block of embryogenesis between the 8-cell and the 16-cell stages (65). Peng H et al. reported that NLRP5 was required for preimplantation embryo development in cows, and NLRP5 knockout embryos were mainly arrested between the 2-cell and 8-cell stages (66). A recent report revealed that mutations in NLRP5 gene in human caused an early embryonic arrest (67).

NLRP5 mutations were shown to be associated with total fertilization failure after IVF/ICSI attempts. To date, two studies reported three mutations associated with fertilization failure (33, 68). In this regard, an autosomal recessive inheritance was suggested according to family pedigree. These identified mutations were missense and frameshift. The two missense variants (c.1598G>C and c.1919T>G; p.Arg533Pro and Leu640Arg), respectively located within the NACHT domain and LRR domain, are predicted to affect NLRP5 protein function, consequently, lead to total fertilization failure in IVF and ICSI cycles. Further studies are still required to reveal NLRP5 function in fertilization as well as its disruption effect on fertilization failure.

### ACTL9 and ACTL7A

ACTL9 (Actin-like 9) [MIM: 619251] and ACTL7A (Actin-like 7A) [MIM: 604303] belong to the family of actin-related genes. They encode actin-like protein 9 and actin-like protein 7A, respectively. ACTL7A is an important paralog of ACTL9. ACTL9 and ACTL7A proteins are co-localized in the sub-acrosomal layer of perinuclear theca (PT) in sperm and interact with each other to maintain acrosomal anchoring to the nuclear membrane.

Two missense and one nonsense mutations in the ACTL9 gene were identified using WES and Sanger sequencing in three individuals with total fertilization failure or poor fertilization in IVF and ICSI attempts (34). ACTL9-mutated individuals exist a higher proportion of tapered-head sperm compared to those in fertile individuals. The sperm acrosome of affected individuals had an abnormal ultrastructure using transmission electron microscopy. These ACTL9 mutations were proved to weaken or lost the ability to interact with ACTL7A, resulting in the inner acrosomal membrane’s detaching from the nuclear and forming a loosened perinuclear theca structure. Mutant sperm show PLCZ1 absence or abnormal localization, which leads to failure to stimulate Ca^2+^ oscillations (34).

Two compound heterozygous variants in ACTL7A were identified in a family with total fertilization failure after ICSI. Pedigree analysis indicated a recessive pattern of inheritance (36). The nonsense variant located in exon 1 of ACTL7A causes a premature stop codon and is predicted to be disease-causing. The missense variant is predicted to be damaging and disease-causing by using a silicon tool. The ultrastructure of the mutation sperm shows defects in the acrosome and perinuclear theca (36). The ACTL7A knock-in mouse model showed reduced expression and abnormal localization of PLCZ1, which could be responsible for the fertilization failure (35).

### ZP2

Zona pellucida (ZP) is an extracellular glycoprotein matrix that surrounds oocytes and medicates several important roles in the acrosome reaction induction, blocking of polyspermy, and protection for the preimplantation embryo (69). ZP2 (zona pellucida glycoprotein 2) [MIM: 182888] encodes the ZP2 glycoprotein, which is a component of ZP composed of four glycoproteins (ZP1, ZP2, ZP3, and ZP4). ZP2 expression in humans was observed in oocytes and granulosa cells as early as the primordial follicle stage (70). Earlier studies on human ZP glycoproteins around the oocyte revealed that unlike ZP1, ZP3, and ZP4, ZP2 mainly binds to acrosome-reacted sperm and the N-terminus of ZP2 mediates the taxon-specific sperm-oocyte binding (71).

The human ZP2 gene, located on chromosome 16, has 19 exons and encodes a polypeptide of 745 amino acids (aa) (72). Two homozygous truncating pathogenic variants of the ZP2 gene were identified using Sanger sequencing in infertile women with IVF fertilization failure (73). The two identified variants were missense and frameshift respectively. Based on the family pedigree analysis, an autosomal recessive genetic mode of infertility was suggested. It was observed that oocytes from affected women had an abnormal ZP with a thinner matrix, and an enlarged perivitelline space compared with normal oocytes. By using transmission electron micrographs and polscope images, there was only one thin layer in the zona matrix of patient oocytes with an irregular network of filaments with large holes (73). The loss-of-function variants of the ZP2 gene led to defective ZP in gamete recognition. In conventional IVF, none of the oocytes were fertilized.

### TLE6

TLE6 (Transducin-like enhancer of split 6) [MIM: 612399] is an effective gene that encodes the TLE6 protein, an essential member of the subcortical maternal complex (SCMC). The TLE6 gene is expressed only in ovaries as well as the oocytes and preimplantation embryos (37). By combining with a variety of maternal effector proteins, TLE6 protein forms SCMC has been linked to key processes occurring during the oocyte-to-embryo transition: cytoskeleton reorganization, meiotic spindle formation, and positioning, organelle redistribution, and cell division (37). Functional knockout studies of the TLE6 gene in mice showed the termination of embryo cleavage as the most phenotype (74). Several mutations within the TLE6 gene were shown to be associated with low-quality embryos (39, 75, 76). In 2015, a homozygous substitution (c.1529C > A) in TLE6 was verified by whole-exome sequencing in three infertile women (77). In 2020, a homozygous missense mutation (c.1226G>A (p.Arg409Gln) in exon 13 of TLE6 was found to be responsible for recurrent total fertilization failure in ICSI cycles (75). According to the in silico computational algorithms, this mutation is predicted to be probably damaging (75). A recessive inheritance pattern was suggested based on family pedigree analysis. Correspondingly, further studies are still required to reveal TLE6 function for the fertilization process.

### DNAH17

The axoneme is the core structure of sperm flagellum and comprises an intricate network of protein complexes, where a central pair of microtubules is surrounded by nine peripheral microtubule doublets (MTDs) in the fixed order (“9 + 2” pattern). Axonemal dynein consist of the outer and inner dynein arms (ODAs and IDAs, respectively). Each dynein arm is a multi-protein ATPase complex, which is composed of light, intermediate, and heavy chain proteins. DNAH17 (Dynein axonemal heavy chain 17) [MIM: 610063], belonging to a member of the dynein axonemal heavy chains (DNAHs) family, encodes a heavy chain protein of ODAs. DNAH17 is mainly expressed in the testis and sperm. Previous reports had revealed that the biallelic mutations of DNAH17 in human sperm were associated with impaired motility and multiple morphological abnormalities of the flagella (MMAF), a rare type of asthenozoospermia which is characterized by absent, short, bent, coiled, and irregular flagella (38, 78–80). Noticeably, the knockout of DNAH17 in mice resulted in morphologically abnormal spermatozoa, showing a phenotype similar to a typical human MMAF phenotype (79).

ICSI is an effective technique used to treat infertility related to MMAF. However, two novel compound heterozygous mutations within the DNAH17 gene were identified using whole exome and Sanger sequencing in an infertile man with markedly diminished sperm motility and caused TFF after two ICSI attempts (81). The nonsense mutation results in a premature stop codon, which leads to a truncated and nonfunctional protein lacking all ATPase domains as well as a microtubule-binding region. The missense mutation was absent in population databases (EXAC, GNOMAD, 1000 Genomes Project) (81). Based on the family pedigree analysis, an autosomal recessive inheritance pattern was suggested. Briefly, mutations in DNAH17 are one of the sperm-related gene mutations associated with TFF.

### Identification and treatments for sperm- or oocyte-borne activation deficiency

Human oocyte activation is characterized by a two-step pattern, called the trigger and oscillator (40). During natural fertilization, the trigger originates from the receptor-mediated interaction between the sperm and the oocyte plasma membrane. During ICSI, the trigger is replaced by a calcium influx called a ‘pseudotrigger’ generated by the injection procedure (40). The actions of the oscillator are described by a series of shorter calcium transients of high amplitude, resulting from the release of sperm-associated factors into the oocyte cytoplasm. The main cause of fertilization failure after IVF/ICSI is considered to be oocyte activation deficiency (OAD), which can be categorized into two kinds: oocyte-borne and sperm-borne activation deficiencies.

Several studies have tried to overcome fertilization failure through a variety of assisted oocyte activation (AOA) methods, including physical, mechanical, or chemical stimuli (82, 83). The application of ICSI combined with AOA improves fertilization rates in the majority of patients with ICSI failure (82, 83). However, not all patients benefit from AOA (84). Many reports have failed to identify which patients are candidates for AOA (85). Some cases of fertilization failure are not solely related to sperm-related OAD and may not require AOA treatment. Therefore, it is important to determine whether AOA is really necessary.

To differentiate between sperm-related OAD and oocyte-related OAD, the mouse oocyte activation test (MOAT), a heterologous ICSI model, can be used as a diagnostic tool (86). The sperm from patients was injected into mature mouse oocytes. Oocyte activation is assessed by examining the percentage of 2-cell formation. When not activated, sperm deficiency is assumed; otherwise, it is suspected that the oocyte is deficient. According to the results of the MOAT, AOA methods can be proposed to correct clear sperm-related OAD (84, 86). Due to inevitable limitations in the heterologous ICSI model, such as multiple steps, long time, and lack of reliability, the PLCZ1 screening assay was recommended to evaluate an oocyte- or sperm-related OAD by a recent study (87).

High throughput sequencing techniques have helped discover some causative gene mutations in infertile couples related to male or female factors associated with poor or failed fertilization after IVF/ICSI attempts. In recent years, a series of mutations in *PLCZ1*, *ACTL7A*, *ACTL9*, *DNAH17*, *WEE2*, *TUBB8, NLRP5, ZP2*, and *TLE6* genes have been identified as potential markers for evaluating fertilization failure in humans (**Table 2**). **Table 3** presents that the use of AOA greatly improves fertilization rates in human cases with sperm-related mutations. However, the AOA method cannot effectively rescue the phenotypes of fertilization failure in cases with oocyte-related mutations (21, 22, 26, 32).

The reasons for fertilization failure caused by oocyte-related defects are often complicated and not easily accepted. Little is known about how to treat oocyte-borne oocyte activation failures. Tesarik J et al. reported that sperm-borne and oocyte-borne oocyte activation failures could be overcome by the use of a modified ICSI technique (40). Heindryckx B et al. recommend AOA as an efficient treatment option in cases of both oocyte- or sperm-related fertilization failure (84). However, these studies mainly focused on case reports. In a recent study, which included 114 patients with a history of extremely poor or complete fertilization failure, the modified stimulation protocol improved clinical outcomes in patients with an oocyte-related OAD (87). A large-sample investigation is needed to support the results.

## Conclusions

The increasing number of IVF/ICSI cycles provides a unique opportunity to systematically evaluate the phenotype of fertilization defects. In the present review, we summarized a series of gene mutations related to poor or failed fertilization, mainly based on high throughput sequencing techniques in the past 10 years. In these studies, a portion of patients with sperm-related mutations obtained pregnancies through ICSI with AOA (13, 19, 34, 36, 46, 50). Just one couple carrying DNAH17 mutations achieved a live birth through donor sperm IVF (81). On the contrary, patients with oocyte-related mutations rarely had their babies, and it was reported that only one patient carrying ZP2 mutations gave birth after ICSI treatment. For women carrying the mutations of WEE2, TUBB8, NLRP5, and TLE6, oocyte donation is considered to be an effective strategy. These findings will help to reveal genetic causes and biomarkers behind poor or failed fertilization and provide some guidance for physicians in hereditary counseling and optimizing clinical treatments.

## Author contributions

YMX and KL collected the literature and drafted the original manuscript. XHC and YPX discussed and revised the manuscript. YMX and KL designed, revised, and edited the work. All authors contributed to the article and approved the submitted version.
